# Complement Evasion in *Borrelia* spirochetes: Mechanisms and Opportunities for Intervention

**DOI:** 10.3390/antibiotics8020080

**Published:** 2019-06-13

**Authors:** Jonathan W. Locke

**Affiliations:** Department of Biology, University of New Mexico, Albuquerque, NM 87131, USA; jonathan.locke@unm.edu

**Keywords:** Lyme disease, spirochete, *Borrelia*, PTLDS, antibiotics, PLD, complement, CRASP

## Abstract

Lyme disease (LD) is an increasingly prevalent, climate change-accelerated, vector-borne infectious disease with significant morbidity and cost in a proportion of patients who experience ongoing symptoms after antibiotic treatment, a condition known as post-treatment Lyme disease syndrome (PTLDS). Spirochetal bacteria of *Borrelia* species are the causative agents of LD. These obligate parasites have evolved sophisticated immune evasion mechanisms, including the ability to defeat the innate immune system’s complement cascade. Research on complement function and *Borrelia* evasion mechanisms, focusing on human disease, is reviewed, highlighting opportunities to build on existing knowledge. Implications for the development of new antibiotic therapies having the potential to prevent or cure PTLDS are discussed. It is noted that a therapy enabling the complement system to effectively counter *Borrelia* might have lower cost and fewer side-effects and risks than broad-spectrum antibiotic use and could avert the need to develop and administer a vaccine.

## 1. Introduction

### 1.1. Borrelia and Lyme Disease

**Figure 1:**
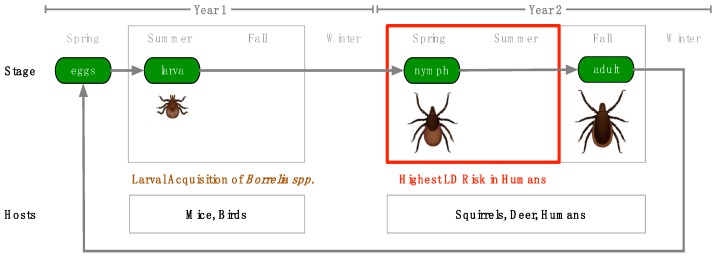
Tick Lifecycle and Host Interaction—The *Ixodes scapularis* tick lifecycle takes two years and requires three blood meals, one at the larval stage, one at the nymphal stage and one at the adult stage of development. Smaller mammals and birds serve as hosts for blood meals at the larval stage, while larger mammals are hosts during the nymphal and adult stages. Larvae which acquire *Borrelia spp.* from mice and birds in the summer of their first year can transmit these spirochetes as nymphs in the spring of their second year, infecting humans. For this reason, Lyme disease (LD) risk is highest in the spring and summer.

Spirochetes of the *Borrelia* genus are corkscrew-shaped, enzootic bacteria endemic in parts of Europe, Asia and North America. *Borrelia* species are fully host-dependent for survival and growth and possess a reduced genome, so this text will refer to the obligate endosymbiotic *Borrelia* as parasites [1]. As parasites of mammals and birds, *Borrelia* bacteria move between hosts, transmitted by tick and louse vectors (Figure 1) [2,3]. A number of *Borrelia* species are known to be pathogenic, and six species are known to cause tick-transmitted Lyme borreliosis in humans, commonly known as Lyme disease (LD): *B. burgdorferi* sensu stricto (s.s.), *B. afzelii*, *B. garinii*, *B. spielmanii*, *B. bavariensis* and *B. mayonii* [4]. In addition, tick-borne relapsing fever (TBRF) is caused by *Borrelia* species, including *B. hermsii*, *B. parkerii*, *B. turicatae, B. duttoni* and *B. miyamotoi*, and louse-borne relapsing fever (LBRF) is caused by *B. recurrentis* (Supplementary Materials, Tables S1 and S2).

#### 1.1.1. Pathogenesis

*Borrelia* spirochetes exist through a complex enzootic cycle that requires them to be well-adapted to tick, mammalian and avian environments. After a tick infected with *Borrelia* takes a blood meal from a host, spirochetes in the midgut migrate through the midgut epithelium into the hemocoel and follow chemotactic clues to locate and infect the salivary glands. Once in the salivary glands, there is opportunity for *Borrelia* to be transmitted through tick saliva to the host [5,6]. Transmission typically takes hours to achieve, but long attachment times are not uncommon as ticks have powerful anesthetic compounds in their saliva, allowing them to feed unnoticed [7]. 

Environmental clues from the vertebrate host, such as the presence of blood-related proteins and an increase in ambient temperature to 37 °C, lead to changes in gene expression, including changes in the expression of surface proteins, that prepare *Borrelia* for the host environment (Supplementary Materials, Table S3) [8]. Once inside the host and after a period of incubation, *Borrelia* disseminates away from the site of infection through use of its endoflagella and by adhering to host cells. Movement away from the site of infection produces the classic erythema migrans (EM) “bullseye” lesion that often accompanies the acute, localized stage of infection. If the infection is left untreated, spirochetes will often disseminate widely throughout the body, resulting in more advanced stages of infection [3]. The pathology of LD appears to be caused primarily by host immune response, as *Borrelia* is not known to produce toxins or proteases that directly damage tissues [9]. The formation of persister cells and biofilms harboring persisters and other microbes [10,11,12,13,14,15,16], as well as other immune evasion mechanisms, likely play roles in pathogenesis and tissue damage through the misdirection of host immune response.

#### 1.1.2. Epidemiology

LD is most common in the northerly latitudes of the Northern Hemisphere, including North America, Europe, Russia, China and Japan. Since 1991, when LD became a reportable condition, records of the incidence and geographic distribution of LD in the United States have increased substantially and incidence in parts of Europe may also be increasing. In part, this increase may be due to improvements in diagnosis and reporting, but cases are also increasing as a result of climate change, which is expanding habitable tick territory and extending time intervals for tick breeding [17,18]. Modeling studies from the Centers for Disease Control (CDC) estimate that there are approximately 300,000 cases of LD per year in the US. These cases are concentrated in a relatively small geographic area. Within the United States, 96% of confirmed cases in 2013 were reported from just 14 states in the Northeast, Mid-Atlantic and Great Lakes regions [19].

#### 1.1.3. Clinical Presentation, Diagnosis and Treatment

The presentation of LD in the early, acute phase of infection is often marked by flu-like symptoms such as fatigue, headache, fever, chills, swollen lymph nodes, myalgia and arthralgia. In some cases, an EM “bullseye” rash may be present as the infection disseminates away from the tick wound site. Within a few days to weeks, the disseminated stage of infection can broaden to include significant neurological symptoms, presenting as Lyme neuroborreliosis (LNB). At this stage, patients may experience migratory pain, facial palsy, acute lymphocytic meningitis, heart block and occasionally, vision problems. In late, disseminated infection, LD can go on to cause persistent arthritis [3].

It is believed that patients that receive early diagnosis and treatment mostly recover following administration of antibiotics (typically doxycycline), but about 10–20% of these patients experience ongoing symptoms, lasting for months to years, known as post-treatment Lyme disease syndrome (PTLDS). The scope of this problem may be still wider as a recent large-scale study revealed that more than 63% of LD cases develop at least one diagnosis associated with PTLDS [20]. Patients who receive late diagnosis and treatment are even more likely to develop PTLDS [21]. The cause of PTLDS is unknown and the subject of ongoing debate. Among the factors that might be involved are autoimmunity, antigenic debris, persistent infection due to antibiotic failure, and tick-borne co-infections such as babesiosis, anaplasmosis, erlichiosis, relapsing fever, tularemia and Rocky Mountain spotted fever (RMSF).

Correct diagnosis and timely treatment of LD can be complicated in a clinical setting by a number of factors, increasing the chance of patients developing PTLDS. As mentioned above, the classic presentation of LD after a tick bite is the EM “bullseye” rash. Unfortunately, patients often do not recall tick bites, and may present either without a rash at all or with lesions that are not easy to identify. In the absence of these textbook indicators, the diffuse symptom picture of LD can mimic a wide range of illnesses, leading to frequent misdiagnosis or non-diagnosis. The difficulty of diagnosis is further increased when the symptom picture becomes more complicated in late disseminated Lyme or when it is clouded by symptoms caused by other tick-borne co-infections, such as those listed above. Even when LD is suspected, false-negative ELISA and Western blot (IgM and IgG) antibody test results are common and can further delay the correct diagnosis [22].

#### 1.1.4. Impact

Within the United States, LD is the most common and fastest growing vector-borne infectious disease, causing significant, long-term morbidity in the minority of patients with PTLDS who do not respond well to antibiotic treatment [23,24] as well as occasionally causing mortality [25,26]. Due to challenges with testing sensitivity and clinical diagnosis, the true number of annual LD infections in the US is likely significantly higher [22]. Medical costs for treatment of LD alone are estimated to be between US$712M and $1.3B per annum [20].

### 1.2. Immune System and Treatment Interactions 

#### 1.2.1. Immune Response 

Normal immune response to *Borrelia* bacteria introduced by tick bite begins with components of the innate immune system. Constitutively expressed antimicrobial peptides and lysozyme at the site of infection directly combat the pathogen, while complement factors opsonize bacteria to enable more efficient phagocytosis and to serve as anchors that enable the formation of membrane-lysing attack complexes. After this initial, direct chemical response, resident antigen presenting cells begin to release cytokines that promote inflammation and chemokines that attract neutrophils, which exit the bloodstream and migrate to the infection site. Monocytes arrive soon after and differentiate into macrophages and dendritic cells. Finally, the adaptive immune response begins to take shape. Antigen presenting cells endocytose bacterial peptides and present them to B cells and T cells, which undergo receptor recombination, maturation and clonal selection. T cells produce cytokines and activate macrophages while B cells secrete highly specific antibodies that bind bacterial surface proteins. In most murine species and natural reservoir hosts, host immune response eliminates obvious objective symptoms. However, this response is insufficient to clear the infection, which then becomes persistent [27]. In humans, there is evidence for asymptomatic infection [28]. It is not known how often *Borrelia* infections are cleared in humans and this data is unlikely to become available in the future as it would be unethical not to treat a human with definite evidence of borreliosis. 

#### 1.2.2. *Borrelia* Antibiotic Evasion and Tolerance Mechanisms 

*Borrelia* has mechanisms to both avoid and tolerate antibiotics. *Borrelia* biofilm likely restricts antibiotic penetration, blocking antibiotics from reaching bacteria residing in the extracellular matrix (ECM) through the binding of antimicrobials to ECM components as well as the inactivation of antimicrobials by enzymes present in the ECM. Intracellular localization represents another strategy to avoid contact with some antibiotics. When *Borrelia* does directly encounter antibiotics, it possesses a non-specific RND type efflux pump that can eject a wide array of antibiotics [29]. If the antibiotic attack is too strong, *Borrelia*’s “stringent response” to environmental stressors is triggered through a RelA/SpoT homolog and the bacteria shift to a highly drug-tolerant persister form. *Borrelia* also forms persister cells stochastically, which ensures that some individuals will survive sudden chemical attacks [11,14].

#### 1.2.3. *Borrelia* Immune Evasion Mechanisms 

**Figure 2:**
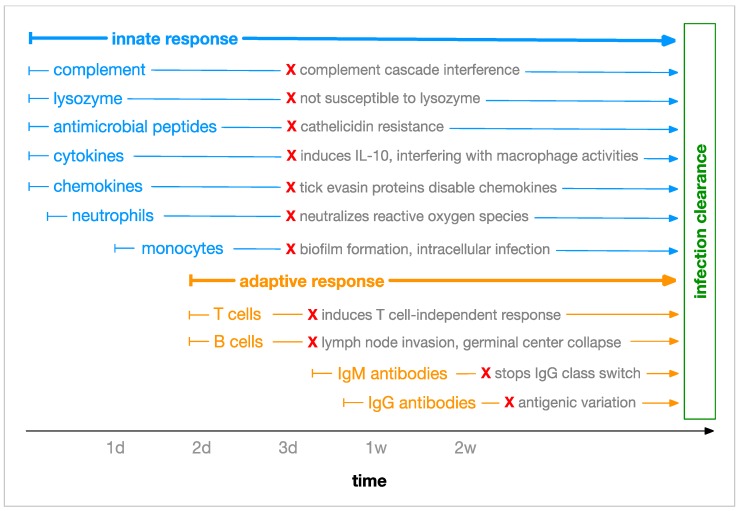
Borrelial Counteractions to Immune Response During Cutaneous Invasion—*Borrelia* evasion mechanisms counter both innate and adaptive immune responses in order to establish persistent infection in the immunocompetent vertebrate host.

To establish and maintain persistent infection in an immunocompetent vertebrate host, *Borrelia* possesses a spectrum of mechanisms which it has evolved in order to evade or otherwise overcome the innate and adaptive immune responses (Figure 2). 

Upon entering the vertebrate host, *Borrelia* spirochetes encounter lysozyme and antimicrobial peptides such as defensin, cathelicidin and cecropins. *Borrelia* has only limited susceptibility to lysozyme and is highly resistant to cathelicidin, although the precise mechanism of this resistance is unknown [27,30]. 

*Borrelia*’s interference with cytokines and chemokines includes two especially interesting mechanisms. Upon entering the vertebrate host, *Borrelia* is assisted in surviving by the tick vector, as tick saliva contains a chemokine-inhibitory evasin protein that serves to reduce the migration of immune cells to the site of infection [31]. When *Borrelia* does encounter macrophages, it stimulates them to produce the anti-inflammatory interleukin, IL-10, which reduces phagocytosis and the expression of MHC II and co-stimulatory receptors in antigen-presenting cells [32].

When professional phagocytes, such as fast-responding neutrophils, arrive at the site of infection, they generate a “respiratory burst” of reactive oxygen species (ROS) including superoxide, peroxide and hydroxyl radicals as well as reactive nitrogen species (RNS) including nitric oxide and peroxynitrite. These chemicals are directly microbicidal but are also known to mobilize other antimicrobials [33,34]. *Borrelia* utilizes a manganese superoxide dismutase (MnSOD) to scavenge superoxide. Different types of SOD enzymes exist, each with unique requirements for metal cofactors such as manganese, copper, zinc and iron. While some MnSOD enzymes are able to function with either iron or manganese as a cofactor, *Borrelia* MnSOD specifically requires manganese, and is in fact not functional when bound to iron [35,36]. The use of manganese makes *Borrelia* independent of iron, which is tightly controlled during infection to prevent microbes from utilizing the resource [37]. However, the exclusive nature of this usage makes *Borrelia* dependent on manganese uptake, which it achieves through *Borrelia* metal transporter A (BmtA) [38]. A notable experiment blocked the BmtA transporter with microbicidal effect, demonstrating that a functioning BmtA transporter is required for survival of the bacteria [39].

To physically evade phagocytes, other immune cells, immune chemicals and antibiotics, *Borrelia* species generate biofilms. The construction of biofilms by *Borrelia* has been demonstrated both in vitro and in vivo in human Borrelial lymphocytomas [12,40]. Another physical strategy used by *Borrelia* to evade immune response is intracellular localization within human endothelial cells. The mechanism for this invasion is partly understood and requires host integrins and Src kinase activity [41].

**Figure 3:**
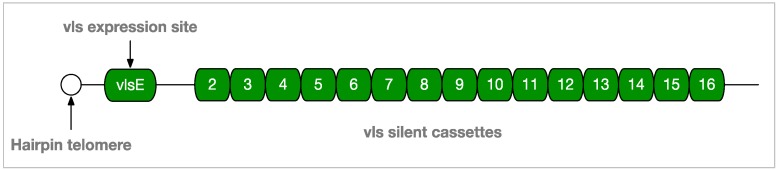
*vlsE* and Silent Cassettes on Linear Plasmid 28-1 of *B. burgdorferi—Borrelia* evasion mechanisms counter both innate and adaptive immune responses in order to establish persistent infection in the immunocompetent vertebrate host. Random segments of silent cassettes 2–16 are recombined at the *vlsE* expression site near the end of lp28-1 to produce surface proteins with a high degree of antigenic variation.

To combat the emerging adaptive immune response, *Borrelia* invades lymph nodes where it skews the adaptive response to a B cell response that is T cell-independent. *Borrelia* also interferes with class switching from IgM to IgG and causes germinal center (GC) “collapse” by altering lymph node structures and inhibiting the development of memory B cells and plasma cells [42,43]. To evade the effective binding of antibodies to its cell surface, *Borrelia* employs an elaborate antigenic variation mechanism. The variable major protein-like sequence expression site (*vlsE*) on lp28-1 (GenBank accession AE000794.2), is recombined through an unknown mechanism involving a Holliday junction helicase, RuvAB, to produce a variable surface protein, VlsE1 (Figure 3). This mechanism is known to be required for long-term survival, is up-regulated soon after infection, and likely produces substantial diversity of surface epitopes by the time an adaptive response is mounted [44,45].

In addition to the immune evasion mechanisms listed above, *Borrelia* has evolved multiple mechanisms to interfere with the complement system so as to avoid opsonization and subsequent phagocytosis or complement-mediated lysis, including mechanisms that capture complement regulatory proteins as well as mechanisms that directly interfere with molecules involved in the complement cascade [4]. These mechanisms and their significance are the subject of this paper and are discussed in detail below.

## 2. Results

### 2.1. Research Overview

#### 2.1.1. Quantitative Analysis of *Borrelia* Research

**Figure 4:**
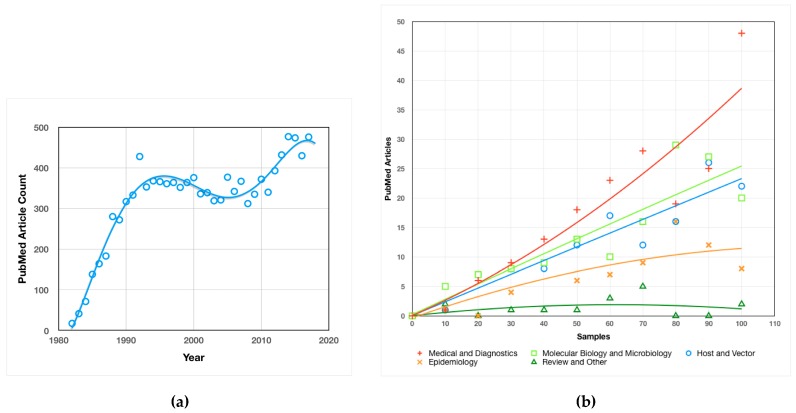
Analysis of *Borrelia* Research: **(a)** *Borrelia* PubMed Article Count by Year—Number of PubMed articles found for the query “*Borrelia*” plotted by year of publication. Curve fitting was achieved by the application of a third order polynomial trendline in the Mac Numbers OS/X application; **(b)** *Borrelia* Research Emphasis—Rarefaction curves indicating the richness of diversity for broad categories of *Borrelia* research.

Research on *Borrelia*, as measured by articles published on PubMed, has trended up since its discovery as the causative agent of LD in 1982, with a brief decline in the early 2000s (Figure 4a). The maturation of initial, basic LD research is a likely reason for the decrease in interest. The considerable increase in research interest in recent years is most likely due to the growing impact of LD as a result of the dramatic, climate change-fueled expansion discussed above.

The emphasis of PubMed-accessible research has been primarily on medical and diagnostic research, with a strong secondary emphasis on molecular and microbiological research, as well as host and vector studies (Figure 4b). Epidemiological studies comprise a somewhat smaller portion of the body of research, and review articles make only a minor contribution to the corpus. As with *Borrelia* research as a whole, the heavy emphasis on medical and diagnostic research is likely a reflection of the increasing prevalence and impact of LD.

#### 2.1.2. *Borrelia* Genomics

The complete genome of *Borrelia burgdorferi* has been determined and its 911 kbp chromosomal genome is available on GenBank (accession NC_001318.1). *B. burgdorferi* (s.s. strain B31-GB) additionally possesses 12 linear and 10 circular plasmids, placing its genome among the most complex bacterial genomes known [46]. Complete knowledge of the *Borrelia* genome, as well as available proteomic (Uniprot) data and other “omics” data, can be expected to drive new research.

#### 2.1.3. Analysis of *Borrelia* Immune Evasion and Persistence Research

**Figure 5:**
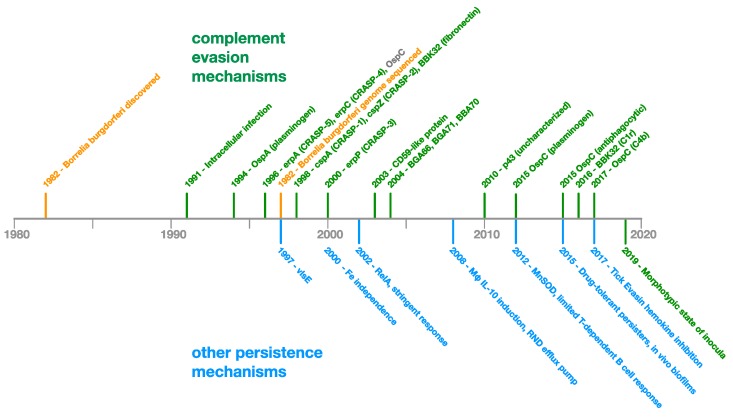
*Borrelia* Immune Evasion and Persistence Research Timeline—A selection of novel immune evasion and persistence discoveries since 1982.

The pace of discovery of immune evasion and persistence mechanisms of *Borrelia* has been relatively constant since 1991, indicating that this area of inquiry is still expanding and further novel discoveries can be expected in the future (Figure 5).

#### 2.1.4. Relative Importance of Evasion and Persistence Mechanisms

The immune evasion and persistence mechanisms shown in Figure 5 are likely all of some importance to the establishment of persistent infection in an immunocompetent host. However, three mechanisms in particular stand out. (1) In vivo biofilms are very likely to be of major importance as they provide a safe, low-oxygen habitat for optimal spirochetal development, allowing nutrients in, while restricting access to phagocytes and most likely to at least some antibiotics. (2) Drug-tolerant persisters are also likely to be of major importance in LD treatment as all antibiotics tested and most antibiotic combinations are not capable of eradicating persister populations [11]. (3) Complement evasion mechanisms have a unique and important role because complement activation via the alternative pathway (AP) and lectin pathway (LP) begin at the moment of infection and continue through activation of the classical pathway (CP) and beyond (see background on the complement system below). Since complement is a threat to *Borrelia* throughout the infectious process, restoration of proper complement function represents a treatment opportunity applicable to all stages of infection, possibly including PTLDS. This review will describe complement function, the current status of *Borrelia* complement evasion research and possible avenues for the development of novel complement-related therapies.

### 2.2. The Complement System

#### 2.2.1. Opsonization

**Figure 6:**
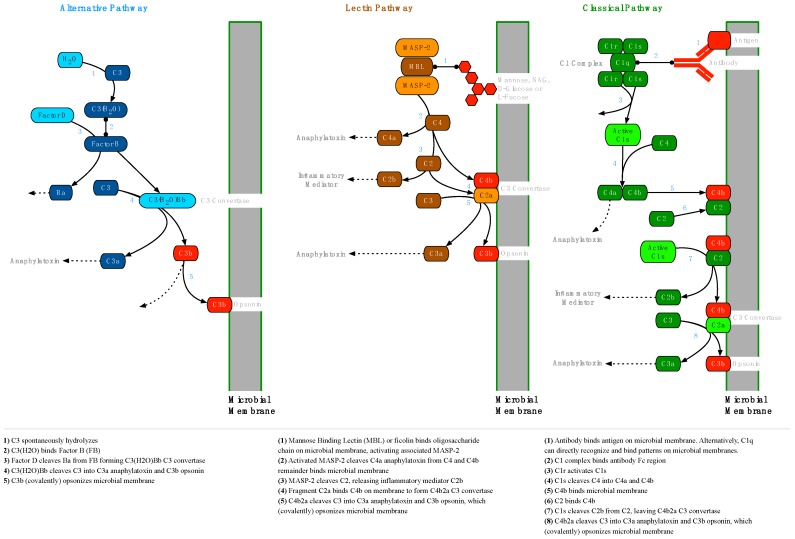
Microbial Opsonization via the Alternative, Lectin and Classical Pathways—To establish infection in a vertebrate host, *Borrelia* must first overcome the constitutive alternative and lectin pathways, and later the classical pathway. The initial stages of each complement pathway leading to (covalent) opsonization by C3b (shown here) are unique, but then converge into a single common pathway to form a membrane attack complex (Figure 7) [37].

The human innate immune system includes an enzyme cascade, known as the complement system, which serves to modulate inflammation, opsonize the cell membranes of pathogens for easier phagocytosis and establish membrane attack complexes (MAC) that lyse pathogens (Supplementary Materials, Table S4). Three separate pathways, the alternative pathway (AP), the lectin pathway (LP) and the classical pathway (CP), converge on the opsonization of the pathogen’s cell membrane with C3b (Figure 6). Opsonization occurs when C3 is cleaved to form C3b, exposing a highly reactive thioester group which forms covalent bonds with hydroxyl and amine groups on cell surfaces.

Once C3b is established on the microbial cell membrane, the soluble factor B (FB) fragment Bb can bind to C3b, forming the AP C3 convertase, which can cleave C3 to create still more C3b. Through this amplification process, the target cell is extensively opsonized. Since opsonins are normally cleared from host cell membranes by complement regulatory factors, the presence of opsonins on cell surfaces enables the recognition of opsonized cells as non-self [37].

#### 2.2.2. Membrane Attack Complex Formation

**Figure 7:**
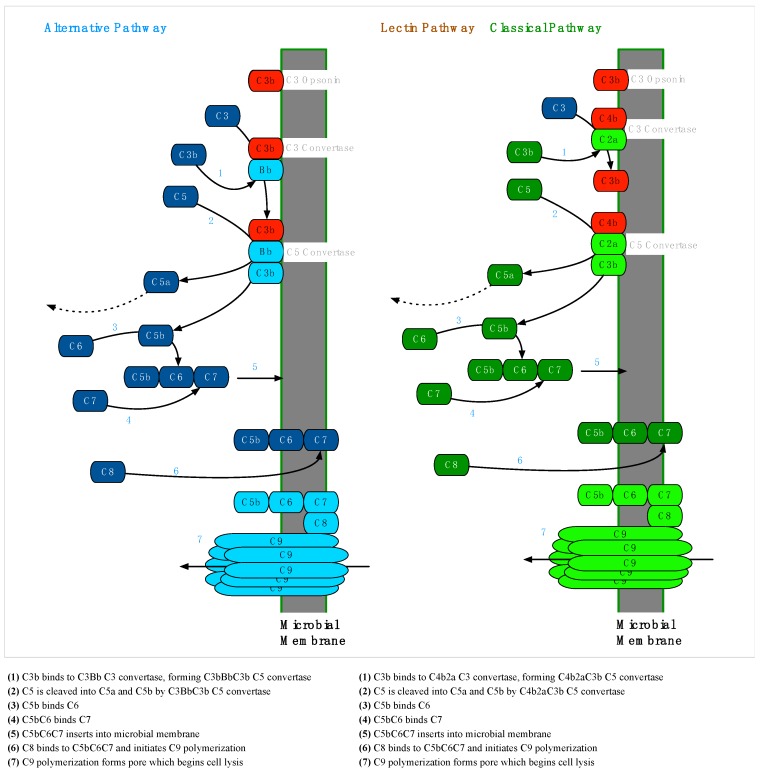
Membrane Attack Complex Formation via the Alternative, Lectin and Classical Complement Pathways—The formation of membrane attack complexes (MACs) is initiated when a membrane-bound C5 convertase cleaves soluble C5 into C5a and C5b. C6 and C7 bind C5b and the resulting C5bC6C7 complex, which is hydrophobic at the C7 end, embeds in the membrane. The MAC polymerizes when C8 binds C7, which induces the addition of several C9 molecules, forming a pore with a hydrophilic center that begins to lyse the cell. C5 convertases of the alternative pathway are formed by an additional C3b molecule joining a membrane-bound C3bBb C3 convertase, forming C3bBbC3b. In the lectin and classical pathways, the C3 convertase C4b2a is joined by C3b to form C4b2aC3b, which serves as the C5 convertase [37].

MAC formation via the terminal pathway (TP) begins when the C3 convertase of any pathway is bound by C3b to form a C5 convertase. Soluble C5 is cleaved to form C5b, which is joined by C6 and C7 to form a C5bC6C7 complex with an exposed hydrophobic region at the C7 end. The hydrophobic end embeds in the microbial membrane, and when C8 binds to this complex, a polymerization reaction is triggered, binding multiple C9 proteins to form a porin channel through the membrane. This channel is hydrophilic on the inside and cellular lysis begins as water exits through the porin (Figure 7) [37].

#### 2.2.3. Complement Regulation

**Figure 8:**
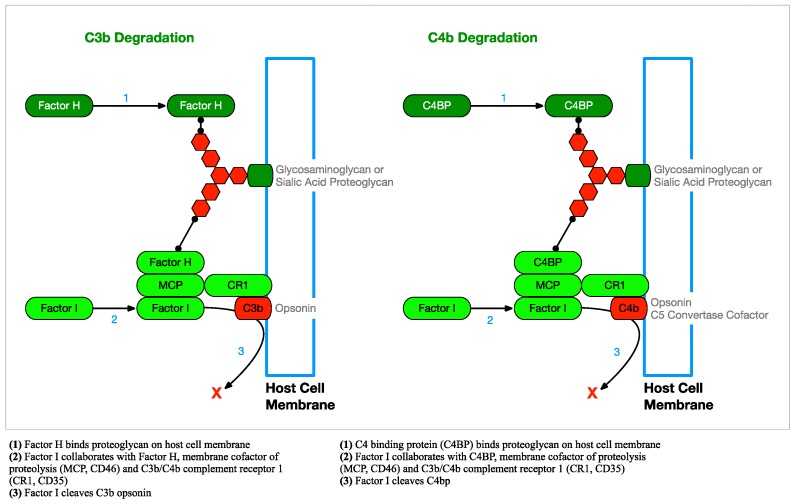
Host Cell Regulation of C3b and C4b Opsonization—Mammalian host cells must be able to degrade C3b (an opsonin) to prevent phagocytosis and C4b (a C5 convertase cofactor) to prevent membrane attack complex (MAC) formation [37]. *Borrelia burgdorferi* has been able to co-opt this regulatory system, avoiding phagocytosis by binding Factor H (FH) with complement regulator acquiring surface proteins (CRASPs) expressed on the bacterial surface.

Because opsonization of cell membranes with C3b happens indiscriminately and it can lead to phagocytosis or lysis, it is necessary for host cells to regulate this activity by actively removing C3b and C4b opsonins. C3b is removed when Factor H (FH) is acquired on the cell surface by binding sialic acid (SA) or glycosaminoglycan (GAG) proteoglycans and cooperates with Factor I (FI) and membrane cofactor of proteolysis (MCP) to cleave C3b bound by C3b/C4b complement receptor 1 (CR1). In a similar fashion, C4 binding protein (C4BP) is acquired and works with FI and MCP to remove C4b bound to CR1 (Figure 8).

**Figure 9:**
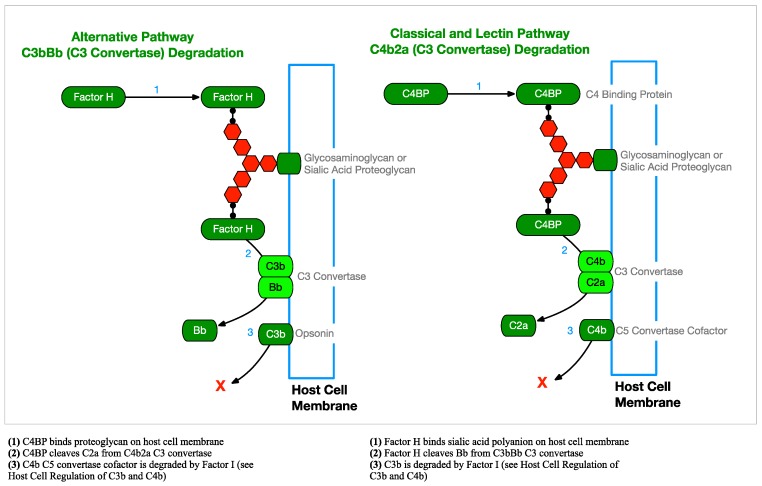
Host Cell C3 Convertase Regulation—Mammalian host cells must be able to degrade the C3 convertases of all three complement pathways to prevent host cell opsonization from potentially leading to phagocytosis [37].

Host cells are also able to remove established C3 convertases. FH binds SA or GAG proteoglycans on the cell surface and cleaves Bb from AP C3bBb C3 convertases. In a similar way, C4BP binds proteoglycans on the cell surface and cleaves C2a from CP and LP C4b2a C3 convertases (Figure 9). The remaining C3b and C4b opsonins can then be degraded by FI, as described above and as shown in Figure 8.

### 2.3. Complement Evasion by Borrelia

*Borrelia* species infect ticks by entering in the blood meal during feeding. Complement activation relies on serum proteins and continues to act within the tick vector. *Borrelia* species vary in their ability to survive complement attack, termed “serum resistance”. *B. afzelii* and *B. burgdorferi* strains are strongly serum resistant while *B. garinii* strains are fairly sensitive to serum. However, even in the most sensitive strains, some spirochetes do survive [47]. After feeding, *Borrelia* leaves the midgut and migrates to the salivary glands where it can be transmitted to a new host during the next feeding [48].

The array of surface proteins utilized by *Borrelia* spp. to evade vertebrate complement in the tick vector during blood meals differs from the set of proteins employed during vertebrate infection, and so the expression of these surface proteins changes accordingly during the transition from vector to host [49]. The reason for expression of different complement evasion proteins with similar function in different environments is unknown and puzzling, and deserves further research to determine whether these proteins have physiological optima under the conditions present inside the tick vector and in vertebrates.

The *Borrelia* surface proteins involved in complement evasion fall broadly into two categories, each of which is explored below. The first category includes those proteins which directly interfere with components of the complement cascade. These proteins inhibit the AP, LP or CP by inactivating key pathway components. The second category of complement evasion proteins includes proteins which subvert normal complement regulation mechanisms by acquiring host regulator proteins.

### 2.4. Category I: Direct Complement Interference

#### 2.4.1. Classical Pathway Inhibition by C1r Binding: BBK32

The *B. burgdorferi* surface protein BBK32 (Table 1) has been known for some time to serve as an adhesin to host cells by binding fibronectin found in the extracellular matrix (ECM) of vertebrates [50]. Substantial portions of BBK32, and the fibronectin binding region in particular, are intrinsically disordered, lacking a rigid 3D structure. More recently, BBK32 was found by Garcia et al. (2016) to inhibit the CP by binding the C1 subunit C1r with high affinity (K_d_ = 3.9 nM), inactivating the serine protease function of C1r and halting the CP cascade. The source of this binding was traced to the C-terminal domain and the effectiveness of BBK32 at inhibiting the CP was demonstrated. An amount of 1 μM of BBK32 provided nearly perfect protection against serum complement hemolysis to sensitized sheep red blood cells and inhibition of the CP was found to be dose-dependent, with a calculated IC_50_ value of 110 nM. The binding of C1 by spirochetes was found in this study to be improved by BBK32, which was further found to confer serum resistance when added to a serum sensitive strain [51].

Since BBK32 inhibits the CP and the CP has been shown to kill *B. burgdorferi* independent of antibody binding, it is possible that BBK32 is sufficiently necessary for infectivity that it could be developed into a promising drug target [52]. This notion is supported by a knockout study that found BBK32 mutants to have significantly reduced infectivity, but it is also somewhat tempered by previous studies which have shown other complement components to have a relatively limited role in controlling infection in mice. 

Garcia et al. (2016) note, however, that the direct microbicidal effects of complement may not be the only issue at stake, suggesting that BBK32-driven complement inhibition caused by spirochetes in infected lymph nodes might be responsible for an observed reduction in C4 deposited on follicular dendritic cells (FDC) in lymph node germinal centers (GC), causing low levels of FDC antigen presentation leading in turn to observed but presently unexplained GC “collapse” [51,53]. 

The phenomenon of GC collapse involves the development of short-lived, abnormal GCs that fail to create sufficient numbers of memory B cells and plasma cells. The resulting immunosuppressive effect is observed for months after infection and is not specific to *Borrelia*. GC collapse is likely crucial to Borrelial persistence, but it may have even greater importance in complex LD cases where co-infections are present or in PTLDS [43].

#### 2.4.2. C3b Degradation by Binding Plasminogen

Activation of the zymogen precursor plasminogen forms the blood protein plasmin, which dissolves fibrin clots. It is also known that plasmin inhibits complement by binding and cleaving C3b and C5 [54]. The ability to dissolve fibrin and to regulate complement deposition makes plasminogen an especially attractive molecule for pathogens to acquire toward interference.

Outer surface protein A (OspA), a prior vaccine target, has been known for quite some time to bind plasminogen [55]. Unfortunately, expression of OspA is down-regulated upon passage of spirochetes into the vertebrate host and so OspA would not make a good drug target if the goal is to improve treatment of patients with active early or late infections.

Outer surface protein C (OspC), is up-regulated in *Borrelia* during vertebrate infection and is known to bind plasminogen [56]. Anti-OspC antibodies were able to significantly reduce plasminogen acquisition and wild type *B. burgdorferi* were found to bind plasminogen only if expressing OspC, regardless of other surface protein expression. This finding appears to contradict other studies enumerated in this section that indicate plasminogen binding also by other outer surface proteins and follow-up studies seem required to elucidate the matter.

The *B. burgdorferi* protein BBA70 was found to bind plasminogen with high affinity and inhibits the microbicidal effects of complement by cleaving C3b and C5. BBA70 was not able to bind complement regulators and follow-up in vitro studies as well as in vivo studies need to be performed [57]. For these reasons, BBA70 is not an attractive therapeutic target to improve complement function. 

*Borrelia* species have evolved complement-regulator acquiring surface proteins (CRASPs) which can bind complement regulators, in particular FH and FH-like proteins. CRASP genes have similar function but two different nomenclatures for historical reasons. The *csp* family of CRASP genes encode “conserved signature proteins” while the *erp* family of CRASP genes encode “OspE-related proteins” (Table 2). In *B. burgdorferi*, *B. afzelii*, and *B. spielmanii*, CspA is able to bind plasminogen [58]. The genes *cspZ*, *erpA*, *erpC*, and *erpP* are only known to exist in *B. burgdorferi* and their expressed proteins also bind plasminogen [4,59].

Serum resistance (insensitivity to complement-mediated lysis) is conferred by both CspA and CspZ. CspA interacts with plasminogen and FH/FHL-1, but also with several additional complement components: C7, C8, C9 and MAC [60,61]. The crystal structure of CspA has been determined and the FH binding site has been characterized to a degree [62,63]. Of the known complement-interacting mechanisms, CspA appears to be one of the best understood, although the Önder et al. (2012) result appears to minimize the role of CspA in binding fibrinogen. Unfortunately, this well-developed target is not a good candidate for antibiotic drug development because it is up-regulated in the tick environment (to confer serum resistance during the blood meal), but becomes strongly down-regulated during entry of the vertebrate host [64].

**Figure 10:**
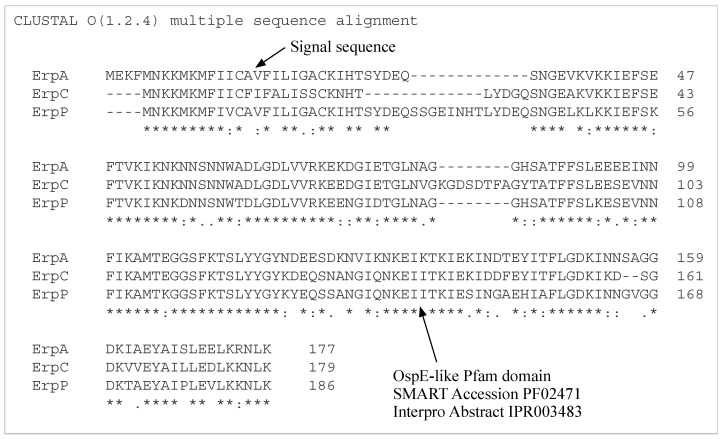
OspE-related (Erp) CRASP Protein Alignment—Clustal Omega (version 1.2.4) alignment for OspE-related (Erp) CRASP proteins shown in Table 2. The region highlighted in green is a shared OspE Pfam domain (SMART accession PF02471, Interpro abstract IUPR003483).

The OspE-related (Erp) CRASP proteins ErpP, ErpC and ErpA have highly similar amino acid sequences (Table 3), while CspA and CspZ are not similar to each other, and neither CspA nor CspZ is similar to ErpP. These differences indicate that the Erp family of proteins share an evolutionary history, while CspA, CspZ and the Erp family of proteins evolved separately. When aligned with Clustal Omega (version 1.2.4), the Erp protein amino acid sequences show similarity, including a common OspE Pfam domain (Figure 10).

#### 2.4.3. MAC Interference

When C3b opsonizes *Borrelia* and direct degradation of C3b opsonins by *Borrelia*-acquired plasminogen and complement regulator proteins fails to remove C3b, *Borrelia* species must prevent the C5 convertase pathway from establishing lytic MAC complex pores on its surface (Figure 7).

*Borrelia* species possess CspA, which is described below (Category II) as interfering with complement regulation. Hallström et al. (2013) discovered that CspA in *B. burgdorferi* has a second function: interfering with MAC formation. CspA binds C7 and C9, inhibiting C9 polymerization and blocking MAC assembly. Transfer of *cspA* by genetic modification to the serum-sensitive species *B. garinii*, caused it to gain serum resistance, indicating that surface expression of the protein is sufficient for MAC inhibition. This discovery was the first of its kind in Gram-negative bacteria [65]. Unfortunately, as noted above, *cspA* expression is down-regulated during entry of *Borrelia* into the vertebrate host such that it does not represent a worthwhile drug target for patients with active infection.

*B. bavariensis* surface proteins BGA66 and BGA71, which have a moderate sequence similarity to CspA, have since been found to inhibit MAC assembly as well and they further inhibit the AP (BGA66), TP (both) and CP (both). Again, transformation of *B. garinii* with these proteins introduced serum resistance [66].

In addition to CspA-mediated MAC inhibition, *B. burgdorferi* also expresses a (human) CD59-like protein on its surface. Blocking this protein with anti-CD59 antibody components removed serum resistance from a normally serum-resistant strain [67]. This protein is not likely to represent a realistic therapeutic target due to its similarity to human CD59 in binding antigen, and it appears that further investigation has not occurred.

### 2.5. Category II: Complement Regulation Interference

#### 2.5.1. Factor H and FHL-1 Binding

In *B. burgdorferi*, *B. afzelii*, and *B. spielmanii*, surface-expressed CspA binds host complement regulators FH and FHL-1 on the bacterial surface, where these proteins remove C3b opsonins, preventing lysis or phagocytosis. In *B. burgdorferi*, CspZ has a similar function (Table 2). The importance of FH acquisition for bacterial survival has been demonstrated by the induction of high serum sensitivity via knock-out of *cspA* [64]. Unfortunately, while CspA conveys serum resistance in vitro, the mechanism is ultimately not critical for infection in vivo. Woodman et al. (2007) demonstrated this in an elegant experiment where they infected both wild-type (WT) and FH-deficient mice with *B. burgdorferi*. The experiment found no detectable level of FH acquired on Borrelial surfaces in the FH-deficient mice and yet found no quantitative difference in level of infection between the two types of mice [68]. These two apparently contradictory findings need a clear reconciliation through further research.

The OspE-related proteins (Erp) ErpA, ErpC and ErpP collectively bind FH, and augment the serum resistance of OspA mutants modified to overexpress OspE proteins. This modification was further shown to translate into a reduction in complement deposition and MAC formation [69]. The crystal structure of OspE was recently determined, including the binding site for FH. Since OspE is highly similar to ErpA, the structure and binding site of ErpA should be very similar. In addition, other experiments have determined the crystal structures of ErpC and ErpP [70]. Unlike CspA and CspZ, bacterial expression of OspE and related proteins (Erps) are up-regulated in the vertebrate host. 

In addition to lipoproteins, some OmpA-like (a porin-like integral membrane protein found in *E. coli*) outer membrane proteins have been found to acquire FH. A screen of *B. garinii* whole-cell sonicate against human sera identified an FH-binding homologue of BG0407, an unknown putative protein sequence identified in *B. bavariensis* (Table 2) [47]. Follow-up work identified another FH-binding homologue in *B. afzelii*, BafPKo_0408 (also known as BAPKO_0422), and provided evidence that it forms an 8-stranded β-barrel similar to the membrane-spanning domain of OmpA (Table 2) [71]. A homologue in *B. burgdorferi*, BB0405, was shown to be surface-exposed, but while required for infection in mice, did not bind human FH [72]. The difference in FH binding between BB0405 and the other homologues, as well as the reason that BB0405 is required for murine infection in spite of not binding FH are both puzzles that seem to require further investigation and clarification. A similar protein, BB0406, produced by a co-transcribed, paralogous gene, was found along with BB0405 to be immunogenic in non-human primates and antibodies against the two proteins were found to be borreliacidal, although BB0406 was not required for infection [73].

#### 2.5.2. C4b Inactivation by Binding C4BP

The CP was shown to be inhibited (*B. burgdorferi*, *B. garinii*) by the binding of C4BP by a protein known as p43. When joined by FI, the captured C4BP protein was able to inactivate C4b, contributing to serum resistance [74]. Unfortunately, this result was contradicted by other experiments that did not observe C4BP binding, so it remains controversial [4].

## 3. Discussion

### 3.1. Lyme Disease Impact and Medical Response

LD is a widespread and rapidly expanding, emerging infectious disease with significant long-term health consequences in the 10–20% of patients who develop post-treatment Lyme disease syndrome (PTLDS). Additionally, 63% of all LD patients develop one or more PTLDS-associated diagnoses [20,21]. The absence of tissue damage caused by known bacterial toxins or proteases may seem to indicate to some that the immune-mediated damage caused by LD is not significant. On the contrary, patients with PTLDS were more likely to report fair or poor health status than patients with MS, lupus, diabetes, PTSD, stroke or congestive heart failure, and further reported significantly higher fatigue, pain, sleep disturbance, and depression than controls [23,24]. 

The medical cost of LD treatment in the US is estimated to be between $712M and $1.3B per annum [20]. Social costs are also high as LD causes significant disability. PTLDS patients between the ages of 25 and 54 have a 45.9% employment rate, versus 81.0% in the general population, a 43% lower rate. An additional 25% of LD patients have reduced work hours or changed occupations as a result of PTLDS, while 24% receive disability [24]. 

In spite of the clear and urgent need for improved understanding leading to the development of treatments for PTLDS, a viable strategic plan is lacking and National Institutes of Health (NIH) funding for LD greatly trails other diseases on a per-case basis (Table 4). NIH funding for LD is just 1.8% to 3.2% of estimated direct annual medical costs. If allocated the same level of funding on a per-case basis that was allocated to West Nile virus research in 2018 (which affected 2,544 individuals in 2018 or 0.8% of the estimated number of LD cases in the same year), the funding for LD would be $5,306,700,000. It would make sense to prioritize LD research, particularly as it relates to PTLDS, and to greatly increase funding for this costly and unmet medical need.

### 3.2. PTLDS or PLD?

The cause of PTLDS is not known and is the subject of ongoing controversy. Hypotheses regarding the mechanism(s) responsible for PTLDS have included autoimmunity [75,76], the post-infectious persistence of spirochetal antigens [77] and permanent tissue damage. There is a growing body of evidence that at least some cases (12 of 12 cases in [16]) involve persistent infection, with an emerging understanding of the underlying mechanisms that may be responsible for antibiotic treatment failure in LD, including immune evasion, intracellular localization, highly antibiotic-tolerant persisters and biofilm formation in vivo [4,11,12,16,43]. 

The morphological state of inocula (MSI) applied to mice has very recently been shown by Feng et al. (2019) to determine both disease severity and antibiotic treatment response. Three morphotypes were found, each with unique properties, including sensitivity to different antibiotics. Planktonic spirochetes caused the least severe pathology and were easily eradicated by standard antibiotic treatment. Round-body antibiotic-tolerant persisters caused more severe illness and were more difficult to treat. Biofilm aggregates caused the most severe disease and were fully resistant to the currently preferred treatment recommendation of 21 days of doxycycline. The combination of daptomycin, ceftriaxone and doxycycline antibiotics was found to eliminate the infection caused by biofilm morphotype inocula in mice, as determined by ear punch culture [13]. However, this result is unlikely to be definitive as it has been contradicted by in vitro studies that did not observe effective bacterial killing [78,79]. In addition, follow-up testing at 12 months was not performed. Such follow-up would be required to determine clearance of infection, as *Borrelia* infection was documented to rebound after this interval by Hodzic, et al. (2014). In the Hodzic, et al. study, mice treated with ceftriaxone in the same manner as previous studies reproduced the results of those studies, yielding non-cultivable tissues with low and falling copy numbers of *flaB* DNA by qPCR. However, at 12 months, while spirochetes were still non-cultivable, *flaB* copy numbers had rebounded in multiple tissues to nearly match those in the saline-treated control mice. This is an especially important result that deserves close attention, because it demonstrates that treatment-resistant *Borrelia* infection is not inconsistent with past observations of non-cultivability [15]. Even more interesting is the reproduction by this study of low antibody titers observed in past studies. This may be the signature of *Borrelia* immunosuppression (potentially via the BBK32 mechanism proposed by Garcia, et al. and discussed above) rather than infection clearance [51,53]. Further, the similarity in cytokine regulation between the sham-treated mice and the antibiotic-treated mice in this study provides a reasonable explanation for the persistence of some of the symptoms present in PTLDS.

It is unknown how these morphotypes express virulence factors as a mixed infective population. This discovery has produced a model system (MSI) for investigating the morphotypic variation of treatment efficacy for what has been designated as persistent Lyme disease (PLD) [13]. The MSI model is applicable to other persistent, inocula-dependent infections such as *Staphylococcus aureus* [80], which underscores the need to advance this body of research.

### 3.3. Opportunities for Intervention

The impact of PTLDS and the growing body of evidence for PLD as a major contributing factor in its pathogenesis makes it imperative that the search for more effective PLD treatments begins now, even if it is not yet known what proportion of patients might respond to such treatments and what proportion might not respond, having other unknown mechanism(s) involved in their PTLDS pathology. There is a wide spectrum of possible interventions against PLD, including antibiotic combination therapies, targeted small-molecule drugs, anti-biofilm agents, phage therapy, natural medicines and antimicrobial peptides. From this range of approaches, it would be desirable to find a single treatment that is highly effective against *Borrelia* alone, avoiding collateral treatment damage to the microbiome. Ideally, this agent would be fully effective from the moment of initial, acute infection through the end of the course of the illness, no matter how late the diagnosis. Due to a mixed model of infection with planktonic spirochetes, round-body persisters, biofilm aggregates, and intracellular bacteria, it will likely be difficult to effect a cure with a single treatment agent. An alternative to directly combating *Borrelia* in its many forms may be to assist the immune system in clearing the infection. Such an approach will require deeper knowledge of the biology and pathogenicity of *Borrelia* and of its immune system interactions in particular.

#### 3.3.1. Immune System Intervention

The functioning human immune system has a great capacity to combat infection. In the case of *Borrelia*, there are asymptomatic individuals with positive LD serology, which indicates that the human immune system is fully capable of controlling, if not eradicating LD infections [28]. Since *Borrelia* can only persist in the human body due to its repertoire of immune-evasive strategies, the successful inhibition of a strategy required for bacterial persistence would enable the immune system to control or eradicate *Borrelia*.

In this light, *Borrelia*’s interference with the complement system is highly interesting from a therapeutics perspective. Complement is expressed constitutively and is therefore in effect from the moment of initial infection onwards. It is directly microbicidal via MAC-mediated lysis and phagocytosis, but also indirectly microbicidal as complement components serve as signal molecules that shape and direct the immune response against the pathogen.

Any bolstering of complement function is expected to improve immune response. Theoretically, direct, parenteral supplementation of LD patients with complement components is a possible intervention, but this likely would be expensive and impractical. A better approach would be to inhibit *Borrelia*’s complement interference to indirectly recover a higher level of natural complement function. Since interference with aspects of the complement system confers serum resistance to *Borrelia*, blocking such interference could restore immune function enough to permit control or clearance of PLD. Since a drug that targets complement interference by *Borrelia* would be highly specific, this approach might be superior to broad-spectrum antibiotic use with its associated risks and damage to the microbiome, and it would be less expensive than the cost of developing a vaccine (US $200 to $500 million) and administering it indefinitely to a large population [81]. Regardless of approach, continued research is critical to understanding the pathobiology of *Borrelia*, the cause(s) of PTLDS and PLD and to develop a cure.

#### 3.3.2. Desirable Criteria for Complement Evasion Intervention

The research reviewed in this paper represents a starting point for intervening in PLD by inhibiting *Borrelia* complement evasion. There are many interesting areas where this body of research could be extended, but the areas which might benefit patients with active early or late infection, potentially including those with PLD (or who are in danger of developing it), relate primarily to *Borrelia* complement resistance mechanisms that meet the following proposed criteria:

1. The mechanism must be constitutively expressed, or up-regulated in the vertebrate host at or soon after initial infection;
2. The mechanism must be required for infectivity and long-term maintenance of infection;
3. The mechanism should be required by all infective morphological forms of *Borrelia*, including antibiotic-tolerant persister cells and biofilm aggregates;
4. The structure of the molecules involved in the mechanism must be known, including ligand binding sites;
5. The structure of the molecules involved should be unique so that small molecule inhibition is likely to avoid off-target effects that would impact microbiota or the patient.

#### 3.3.3. BBK32

The deposition of complement factor C4 (Figure 6) exemplifies the crucial dual role of complement in controlling infection. C4b plays an important role as an opsonin, but it also stimulates antigen uptake and presentation in follicular dendritic cells (FDCs) in lymph nodes [37,82]. It is logical then, that the inhibition of the CP by the *B. burgdorferi* surface antigen BBK32, would lead to the observed reduction in C4b deposition on FDCs, and that this would lead in turn to reduced FDC antigen presentation and finally to observed GC “collapse”, where short-lived germinal centers in lymph nodes produce inadequate populations of memory B cells and plasma cells [51,53].

The potential ability of BBK32 to suppress antibody response may be directly involved in the establishment of PLD as a chronic infection, as well as in *B. burgdorferi* Western blot antibody test insensitivity observed in LD patients for decades. Because this immunosuppressive effect continues for months after infection and because it limits the ability to produce antibodies against co-infections, the issue is critical for patients who may be infected with many other tick-borne microbes, including *Babesia*, *Anaplasma*, *Erlichia* and *Tularemia.* Without a functioning CP, it is not likely that the immune system can mount an effective response against these organisms [42,43]. Further, the effect of CP inhibition would be synergistic as each co-infecting organism likely has its own spectrum of mechanisms for evading and suppressing the immune system.

Recovery of the CP from inhibition by BBK32 would restore the immune system’s ability to opsonize microbial cells and to generate an effective antibody response. A strong antibody response may enable the immune system as a whole to control or clear the infection, with potential to cure PLD. As an added benefit, a strong antibody response would be easier to detect in diagnostic testing.

Although BBK32 is required by *B. burgdorferi* for optimal infectivity in an animal model, functional characterization has been limited to observation that BBK32 binds fibronectin during early infection [53]. Since it was only recently discovered that BBK32 inhibits the CP by binding C1r [51], it is not known how BBK32 expression affects persistent infection. The MSI model for studying PLD proposed by Feng et al. (2019) would be ideal for studying the infectivity of a BBK32 mutant—as a planktonic spirochete, as a round-body persister and in biofilm aggregate form. The same model could also be used to evaluate the effect of BBK32 on *B. burgdorferi* Western blot test sensitivity, as poor antibody production might increase the rate of false negative diagnoses.

The role of BBK32 in GC collapse needs clarification and might well lead to further insights into PLD and chronic disease. In vivo studies of murine GCs exposed to BBK32 mutant strains and wild-type *Borrelia* species could help clarify this issue. Again, the MSI model could be useful in highlighting any morphotype-dependent differences in the effects of BBK32 on FDCs and GC function.

The initial study by Garcia et al. (2016) of *B. burgdorferi* protein lysates which determined that C1 was being inhibited requires further inquiry. There were additional probe-reactive bands in the Far Western protein blot of BBK32 which need to be investigated to determine if *B. burgdorferi* (or other *Borrelia* species) can express other proteins with BBK32-like functionality.

Although the structure of BBK32 has not yet been fully determined, the crystal structure of the C-terminal binding region for C1r was resolved and published during the writing of this paper, indicating a high interest in this protein [83]. A BLAST search for the *B. burgdorferi* BBK32 protein amino acid sequence revealed no matches outside the *Borrelia* genus. This is encouraging because it indicates that targeting BBK32 is likely to be highly specific to *Borrelia*, avoiding possible collateral damage to the microbiome and other off-target effects. If inhibition of BBK32 binding sites by a small molecule turns out to be effective against antibiotic-tolerant persister cells and biofilm aggregates in vivo, then that molecule might represent a strong target for the development of a drug treatment against PLD.

#### 3.3.4. Erp CRASPs

The function of Borrelial Erp proteins in complement evasion, as well as the availability of crystal structures and binding site information, identifies this protein family as a feasible target for development of antibiotics. However, although serum resistance was observed to be improved by OspE overexpression, the question needs to be raised as to whether this really matters since the Woodman et al. (2007) experiment showed that FH acquisition by *Borrelia* does not necessarily translate into infectivity (although it noted that additional mechanisms may be involved). This issue needs to be resolved before the FH-binding regions of CRASPs could be considered an attractive drug target.

Although the Woodman et al. study (2007) does cast considerable doubt on FH-acquiring CRASPs being a mechanism required for infectivity, it is possible that ErpA, ErpC or ErpP are required as virulence factors for infectivity in any case, as these CRASPs may have other functionality or they may act in concert with as yet unknown mechanisms. CRASPs are well-studied and characterized, so it would be well worth determining if these OspE-related surface proteins are actually required for infectivity with a study of *Borrelia* sequence mutants in a mouse model. This study would need to incorporate the Feng et al. (2019) MSI model to clarify to what extent each morphotype depends on expression of Erp, as protein expression levels may vary by morphotype.

#### 3.3.5. OmpA-Like OM Proteins

Although the OmpA-like outer membrane protein BB0405 (*B. burgdorferi*) does not bind FH, it is required for infection, and being immunogenic is known to be expressed during mammalian infection. For these reasons, BB0405 and the related FH-binding proteins BG0407 (*B. garinii*) and BafPKo_0408 (*B. afzelii*)) deserve further research as they could represent one or more viable drug targets. The puzzle as to why BB0405 was not found to bind FH while the homologous BG0407 and BafPKo_0408 were found to bind it, needs to be resolved and an understanding of whether these proteins represent a common target or two or more distinct targets needs to be developed. Finally, crystal structure and binding site information would need to be determined to advance these OmpA-like OM proteins as potential drug targets.

#### 3.3.6. Other Targets

The *B. bavariensis* MAC-inhibiting and AP/CP/TP-inhibiting surface proteins BGA66 and BGA71 would ideally deserve further investigation, but the medical value of these proteins is uncertain as *B. bavariensis* is not a high-impact infectious agent and these proteins are species-specific. MAC interference by CspA is quite interesting from a basic research standpoint, but seems unlikely to represent a strong target for drug development due to the down-regulated expression of CspA during pathogenic invasion. Lastly, the CD59-like protein expressed by *B. burgdorferi* is not a likely drug target because it is similar to human CD-59. Any drug that interfered with this protein, as expressed by *B. burgdorferi*, would run a high risk of interfering with CD-59 itself in humans.

## 4. Materials and Methods 

Research articles cited herein are generally either primary research or review articles and were sourced from PubMed (https://www.ncbi.nlm.nih.gov/pubmed/) or University of New Mexico library search. The research rarefaction curve data used to create Figure 4 was compiled by randomly sampling and categorizing 550 of the 12,062 PubMed articles matching the query “*Borrelia*”. Categorization of articles was accomplished primarily by inspection of the title or by reading the abstract. Articles that incidentally used molecular or microbiological techniques to accomplish medical, epidemiological, vector or host study ends were not categorized based on the use of such techniques, but rather on the nature of the study. The immune evasion and persistence research timeline (Figure 5) was constructed by review of the literature. Discoveries included on the timeline were deemed to be novel and significant to bacterial persistence. 

## 5. Conclusions

Chronic and mental health conditions consume 90% of the estimated US $3.3 trillion spent annually on health care in the US (Centers for Disease Control). At this time, there are many urgent questions that need to be answered regarding the potential relationships between chronic infection, such as that found in PLD, and a broad spectrum of chronic conditions. The challenging questions posed by PTLDS and PLD, and by persistent and polymicrobial bacterial infections in general, represent vital new territory for science to explore, and eventually to conquer, delivering a new understanding and new treatment capabilities.

The journey towards understanding and cure of PTLDS and PLD has begun in earnest with the research reviewed in this paper. The understanding that antibiotic-tolerant *Borrelia* persisters, intracellular infection and *Borrelia* biofilm aggregates have important roles in the pathogenesis of PLD and its treatment is a valuable and novel new perspective. The demonstration that the *B. burgdorferi* surface protein BBK32 not only inhibits the CP, but may be further implicated in germinal center (GC) “collapse” and subsequent systemic immunosuppression is especially exciting. Further research into this phenomenon may lead to a clearer, broader understanding of pathogenic persistence and antibiotic treatment failure. As a practical application, the successful inhibition of BBK32 could represent a targeted cure for PLD, avoiding the $200 to $500 million cost of developing a vaccine and the ongoing costs of administering it to a large population [81]. Finally, the very recent report that the characteristics of persistent infection (*S. aureus*—Yee 2018, *B. burgdorferi*—Feng 2019) vary by the morphotypic state of inocula (MSI) is potentially a major development. This finding has the potential to shed light on the intricacies of PLD, providing a yardstick to measure and compare results and a way to prioritize and direct PLD research, and this landmark discovery may well turn out to be important in the exploration of chronic infection in general.

Our understanding of persistent infection and chronic disease processes is still very much in its infancy. While science in today’s “omics” era and beyond can and eventually will help to shed light on the mechanisms involved in complex, chronic infections—such as those in PLD—the urgent need and slow pace of progress beg the question: *how can we improve our approach*?

The appearance of MSI [13,80] as a model to interpret persistent infection is well-timed. It coincides with an increasing interest in complex microbial communities, such as those found in biofilms and the human microbiome, as well as an increasing ability to acquire, process and interpret vast quantities of multidimensional data, combining basic care with traditional and modern research approaches. All this activity together may cause a more nuanced and complex understanding of infection to emerge. One hundred and twenty-nine years ago, Robert Koch’s postulates established for the first time a direct, causative connection between microbe and disease [84]. Although this simple and elegant model has been highly successful, perhaps we are slowly ushering in a “post-Koch” era, where infection is increasingly viewed as having dimensions more complex than the examination of a single infectious microbe in a single morphotypic state in relationship to a single, and typically acute, disease process.

As research continues to progress, our understanding of the mechanisms that *Borrelia* employs in pathogenesis and persistence will yield opportunities in the near future to intervene in PTLDS and PLD. This journey will also create inroads that lead toward a deeper and more nuanced understanding of chronic disease.

## Figures and Tables

**Table 1:** Proteins which interfere directly with complement. GenBank accession numbers are for *B. burgdorferi* strain B31 except for BBA70, which is for *B. burgdorferi* strain 163b.

Protein	Gene	Plasmid	GenBank Accession	Species
BBK32	*bbk32*	lp36	AE000788.1	*B. burgdorferi, B. afzelii, B. garinii*, others
OspA	*ospA*	lp54	AE000790.2	*B. burgdorferi, B. afzelii, B. garinii*, others
OspC	*ospC*	cp26	AE000792.1	*B. burgdorferi, B. afzelii, B. garinii*, others
BBA70	*bba70*	lp54	AY696552.1	*B. burgdorferi 163b, B. bavariensis*

**Table 2:** Complement-regulator acquiring surface proteins (CRASPs) and related FH-binding proteins, gene names, plasmid replicons, base pair intervals and the species in which they occur. The acronym *csp* stands for “conserved signature protein” and *erp* stands for “OspE-related protein” [4]. GenBank accession numbers are for *B. burgdorferi* strain B31 except for *erpC*, which is for strain B31_NRZ.

CRASP	Gene	Plasmid ^1^	Interval	GenBank Accession	Species
CRASP-1	*cspA*	lp54	46473-47228 ^2^	AE000790.2	*B. burgdorferi, B. afzelii, B. spielmanii*
CRASP-2	*cspZ*	lp28-3	2260-2970 ^2^	AE000784.1	*B. burgdorferi, B. afzelii*
CRASP-3	*erpP*	cp32-9	26210-26770	AE001581.1	*B. burgdorferi*
CRASP-4	*erpC*	cp32-2	26834-27373	NZ_CP019757.1	*B. burgdorferi*
CRASP-5	*erpA*	cp32-1	26235-26768	AE001575.1	*B. burgdorferi*
-	*BG0407*	-	417734-418345	AAU07257.1	*B. garinii, B. bavariensis*
-	*BafPKo_0408* (*BAPKO_0422*)	-	419301-419906	ABH01676.1 (CP000395.1)	*B. afzelii*

^1^ lp = linear plasmid, cp = circular plasmid. ^2^ Reverse strand.

**Table 3:** Clustal Omega (version 1.2.4) alignment identity, similarity and gaps for Erp amino acid sequences.

Proteins	Identity	Similarity	Gaps
ErpA vs ErpC	77%	85%	5%
ErpA vs ErpP	75%	83%	6%
ErpC vs ErpP	70%	78%	11%

**Table 4:** In spite of a high annual cost for treatment and significant social costs, NIH funding for LD significantly trails that appropriated for other diseases.

Disease	NIH Funding (Millions, 2018)	US Cases per Year	Funding per Case
Malaria	$202	1700 ^1^	$118,824
HIV/AIDS	$3000	38,739 ^2^	$77,441
West Nile virus	$45	2544 ^3^	$17,689
PTLDS	$23	45,000 ^4^	$511.11
LD	$23	300,000 ^5^	$76.67

^1^ CDC estimate (2018), ^2^ CDC (2017), ^3^ CDC preliminary (2018), ^4^ 15% of CDC estimate (2015), ^5^ CDC estimate, (2015).

## Supplementary Materials

The following are available online at https://www.mdpi.com/2079-6382/8/2/80/s1, Table S1: Major Pathogenic *Borrelia* Species, Table S2: Terms Related to Lyme Disease and Other Borrelial Diseases, Table S3: *Borrelia* Surface Proteins, Table S4: Complement System Terms.
