# Supplementary material for: Complement Evasion in Borrelia spirochetes: Mechanisms and Opportunities for Intervention
Source: Antibiotics (Basel). 2019 Jun 13;8(2):80. doi: 10.3390/antibiotics8020080 (PMC6627623; doi:10.3390/antibiotics8020080)
Supplement: Supplementary file 1 [file antibiotics-08-00080-s001.pdf]

Table S1. Major Pathogenic *Borrelia* Species.

B. afzelii [1]	**Europe (1994), Asia**
B. andersonii	North America (1995)
B. bavariensis	Europe (2009), North America, Asia
B. burgdorferi [1]	North America (1982), Europe, Asia
B. garinii [1]	Europe (1992), Asia, frequent cause of neuroborreliosis
B. mayonii [1]	North America (2016)
B. miyamotoi	Japan (1995), North America (2001), Europe, causative agent of TBRF
B. valaisiana	Europe (1997), Asia

[1] Causative agent of LD.

Table S2. Terms Related to Lyme Disease and Other Borrelial Diseases.

**Borreliosis**	***Borrelia* infection**
CLD	Chronic Lyme disease
Early disseminated LD	Initial dissemination of infection
Early localized LD	Acute phase of infection
Late disseminated LD	Persistent phase of infection
LBRF	Louse-borne relapsing fever
LD	Lyme disease
LNB	Lyme neuroborreliosis
MSI	Morphological state of inocula
Neuroborreliosis	Infection of CNS by *Borrelia*
PLD	Persistent Lyme disease
PTLDS	Post-treatment Lyme disease syndrome
s. l.	sensu lato ("in the wide sense")
s. s.	sensu stricto ("in the strict sense")
TBD	Tick-borne disease
TBI	Tick-borne infection
TBRF	Tick-borne relapsing fever

Table S3. *Borrelia* Surface Proteins.

**BBA70**	***Borrelia* surface protein which binds plasminogen and cleaves C3b and C5**
BBK32	Fibronectin binding protein that inhibits C1r, inhibiting the CP
BGA66	*B. bavariensis* surface protein that inhibits MAC assembly, AP, TP and CP.
BGA71	*B. bavariensis* surface protein that inhibits MAC assembly, TP and CP.
BmtA	*Borrelia* metal transporter A (Mn)
CRASP	Complement-regulator-acquiring surface protein
CspA (CRASP-1)	CRASP (ORF bba68 on lp54), expressed in tick environment. Homodimer binds FH/FHL-1, conveying serum resistance to blood meal via complement deposition evasion.
CspZ (CRASP-2)	CRASP (ORF bbh06 on lp28-3), expressed in vertebrate environment. Binds FH/FHL-1, enhancing serum resistance via complement deposition evasion.
Erp	OspE-related protein
ErpA (CRASP-5)	CRASP, up-regulated during transmission to vertebrate host
ErpC (CRASP-4)	CRASP, up-regulated during transmission to vertebrate host
ErpP (CRASP-3)	CRASP, up-regulated during transmission to vertebrate host
OspA	Outer surface protein A, lipoprotein expressed in tick environment
OspB	Outer surface protein B, lipoprotein expressed in tick environment
OspC	Outer surface protein C, lipoprotein expressed in vertebrate environment
OspD	Outer surface protein D, lipoprotein expressed in tick environment
OspE	Outer surface protein E, lipoprotein expressed in vertebrate environment
OspF	Outer surface protein F, lipoprotein expressed in vertebrate environment
VlsE	Expression region of a VMP-like sequence in *B. burgdorferi* which undergoes recombination to produce antigenic variation
VMP	Variable major protein, recombined antigen in *B. hermsii*

Table S4. Complement System Terms.

**AP**	**Alternative pathway of complement system**
Ba	Factor B fragment a
Bb	Factor B fragment b
C1, C1r, C1s, C2, C3, C3a, C3b, C4, C4a, C4b, C5, C5a, C5b, C6, C7, C8, C9	Complement cascade proteins
C3bBb, C4b2a	C3 convertases
C4b2a3b, C3bBb3b	C5 convertase
C4bp	C4b binding protein
CD59	(Human) MAC inhibitory protein (protectin)
CP	Classical pathway of complement system
CR1	C3b/C4b complement receptor 1
DAF	Decay accelerating factor (CD55)
FB	Factor B
FD	Factor D
FH	Factor H
FHL-1	Factor H-like protein 1
FHR	Factor H-related protein
FI	Factor I
LP	Lectin pathway of complement system
MAC	Membrane attack complex (see TCC)
MASP	MBL-associated serine protease
MBL	Mannose binding lectin
MCP	Membrane cofactor of proteolysis
TCC	Terminal complement complex (see MAC)
TP	Terminal pathway of complement
